# Impurity-Induced Magnetization of Graphene

**DOI:** 10.3390/ma15020526

**Published:** 2022-01-11

**Authors:** Michał Inglot, Tomasz Szczepański

**Affiliations:** Department of Physics and Medical Engineering, Rzeszów University of Technology, al. Powstańców Warszawy 6, 35-959 Rzeszow, Poland

**Keywords:** graphene, magnetism, impurity, localized states, resonant states, spintronics

## Abstract

We present a model of impurity-induced magnetization of graphene assuming that the main source of graphene magnetization is related to impurity states with a localized spin. The analysis of solutions of the Schrödinger equation for electrons near the Dirac point has been performed using the model of massless fermions. For a single impurity, the solution of Schrödinger’s equation is a linear combination of Bessel functions. We found resonance energy levels of the non-magnetic impurity. The magnetic moment of impurity with a localized spin was accounted for the calculation of graphene magnetization using the Green’s function formalism. The spatial distribution of induced magnetization for a single impurity is obtained. The energy of resonance states was also calculated as a function of interaction. This energy is depending on the impurity potential and the coupling constant of interaction.

## 1. Introduction

One of the most interesting aspect of current reserch in graphene-based materials is to understand the magnetic properties in doped graphene. Investigation of electron transport properties and structure of electron orbitals is one of the most important aspects of testing graphene for electronic purposes. Moreover, from the point of view of spintronics applications, research is conducted to determine spin transport and spin polarization capabilities of graphene-based devices. Recent studies of the ferromagnetic properties of doped graphene at room temperatures indicate a great potential electronic application of this material. In parallel with experimental research, it is necessary to gain an in-depth understanding of the theoretical aspects underlying the unique properties of graphene. In our paper, we focused on calculations of magnetization and resonant energy levels of the impurity located at the graphene lattice.

The main objective of this work was to determine the wave function and corresponding eigenenergy of a localized state for the graphene lattice with impurity [1,2]. Such a function is a superposition of the Bessel functions and is the solution of Schrödinger’s equation. The results obtained in frame of the relativistic model of massless fermions in the vicinity of Dirac points are analyzed [3,4]. The localized state solution of Schrödinger’s equation for a single non-magnetic dopant is found and the energy of this state is calculated. These results indicate the presence of some resonance levels depending on the impurity potential [5]. The effect of potential has been considered as a perturbation in the model of Hamiltonian with non-interacting particles.

We also found that impurities induce the magnetization of graphene that can be calculated by introducing the disorder operator into the Dirac Hamiltonian [6]. For the calculation of higher orders of perturbation, the Green’s function formalism can be used. As a result, we determine the local magnetization profile. The spin polarization effect for different values of the interaction coupling constant was included in our calculation and the resonance impurity levels have been determined [7,8]. These results demonstrate the significant effect of impurity potential on the value of magnetization in graphene [9].

Magnetic impurities are responsible for creating a system of isolated magnetic moments in graphene. Magnetic interactions between these moments are depending on their specific location in the graphene sublattices and the density of free electrons (holes). Besides, the particularity of graphene electron structure makes it possible to appear an indirect RKKY interaction in the absence of free carriers through the polarization mechanism of electron-hole system [10]. As a result, magnetic interactions can be responsible for ferromagnetic or antiferromagnetic ordering of the moments [11].

Another mechanism of magnetic ordering leading to possible transition from dominating ferromagnetic to antiferromagnetic ordering has been proposed for the case of graphene nanoribbons [12]. It was describes as a mutual effect of chemical potential and spin–orbit coupling [13]. Corresponding numerical calculations have been done for the model with magnetic impurities located at the zigzag edge of graphene nanoribbon [12].

It was also found that the charge transfer between the electron states localized at the zigzag edge and the magnetic impurity state is important for magnetic properties of graphene. It was shown that this mechanism can lead to the compensation of magnetic moment at the nanoribbon edge [14].

For the case of graphene bilayers with impurities adsorbed on one layer and gated by electric field perpendicular to the layers, one can observe the bound states with energies in the gap [15]. These states in the gap are due to impurities. The structure and localization of these states depend on an internal polarization of graphene [9].

The magnetism of graphene resulting from interaction between transition-metal and carbon atoms can be also analyzed by taking into account the spin polarization of metallic impurities. The calculations of magnetic moments indicate a significant doping-induced magnetization effect [16].

Our article is divided into several sections. In Section 2 (Cethods and Calculations), we analyzed the solutions of the Schrödinger equation for the two-component wave function to determine the energy of the state located on a single impurity in the graphene lattice. The level of magnetization induced by the presence of the dopant was also calculated. Based on a model of the impurity with spin, we obtained the value of the spin projection on the direction determined by the magnetic moment. In Section 3 (Results), we discussed the effect of magnetic correlations on energy levels, and presented the distribution of local induced magnetization as a function of distance from the impurity. In Section 4 (Summary and Discussion), we have addressed the effectiveness of the method used to describe magnetization in doped graphene compared to results of previous work.

## 2. Methods and Calculations

**Figure 1:**
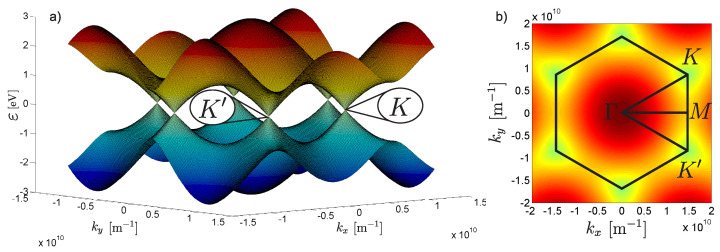
(**a**) First Brillouin zone in graphene with marked points, (**b**) Γ—center of the zone, *M*—center of the edge, *K* and $K^{\prime }$ are the nonequivalent Dirac points.

Most importantly, from the point of view of electronic properties of graphene, is a neighborhood of the Dirac point. Here the energy bands form the Dirac cone, shown in Figure 1. The linear part of the energy spectrum describes weakly excited massless Dirac fermions with certain chirality. In pure graphene, the Fermi level lies exactly at the Dirac point and the massless fermions can be described in the model of (2 + 1) dimensional quantum electrodynamics.

(1) $$i\hbar \frac{\partial \;\Psi (x,t)}{\partial \;t}=\left(c\;\alpha \cdot p+\beta mc^{2}\right)\;\Psi \left(x,t\right),$$

where p=−iℏ∇ is the momentum operator, *m* is the rest mass of electron, *c* the light velocity, and α i β are the matrices 4×4:

(2) $$\alpha =\left[\begin{array}{cc} 0 & \sigma \\ \sigma & 0 \end{array}\right],\;\beta =\left[\begin{array}{cc} I & 0\\ 0 & -I \end{array}\right],$$

σ is the vector, which elements are the Pauli matrices, and I is the identity matrix.

For the wave equation we use the Weyl–Dirac Hamiltonian (WDH), which in the wave vector representation $k=[k_{x},k_{y}]$ has the form of matrix

(3) $$\begin{array}{c} {\hat{H}}_{0,k}=\left(\begin{array}{cc} 0 & vk_{-}\\ vk_{+} & 0 \end{array}\right), \end{array}$$

where $k_{\pm }=k_{x}\pm ik_{y}$ i $k_{x,y}\in BZ$, and BZ denotes the Brillouin zone. As we see from the form of the Hamiltonian Equation (3), its eigenvalues are linear with respect to the wave vector $E_{k}=\pm vs.k$. This implies that the dispersion relation for the unperturbed system near the Dirac point *K* is linear. Similarly, for a Dirac point $K^{\prime }$, WDH can be written as $H_{WD}=\mathrm{vs}.\;\tau^{*}\cdot k$, where $\tau^{*}=\left[-\tau_{x},\tau_{y}\right]$. For the *K* point, we present the wave function of this Hamiltonian in the momentum representation

(4) $$\begin{array}{c} \psi_{\pm ,K}\left(k\right)=\frac{1}{\sqrt{2}}\left(\begin{array}{c} e^{-i\theta_{k}/2}\\ \pm e^{i\theta_{k}/2} \end{array}\right), \end{array}$$

while for the Dirac point $K^{\prime }$

(5) $$\begin{array}{c} \psi_{\pm ,K^{\prime }}\left(k\right)=\frac{1}{\sqrt{2}}\left(\begin{array}{c} e^{i\theta_{k}/2}\\ \pm e^{-i\theta_{k}/2} \end{array}\right). \end{array}$$

The signs “+” and “−”correspond to conduction and valence bands, respectively. The phase factor $\theta_{k}$ describes the relation $\theta_{k}=\tan^{-1}\frac{k_{x}}{k_{y}}$. The wave functions for two non-equilibrium Dirac points *K* i $K^{\prime }$ are connected by time-reversal transformation. Moreover, the points *K* and $K^{\prime }$ connects the reflection operation with respect to the axis $k_{x}$: $\left(k_{x},k_{y}\right)\to \left(k_{x},-k_{y}\right)$ [17,18,19].

### 2.1. Localized States of a Single Impurity

**Figure 2:**
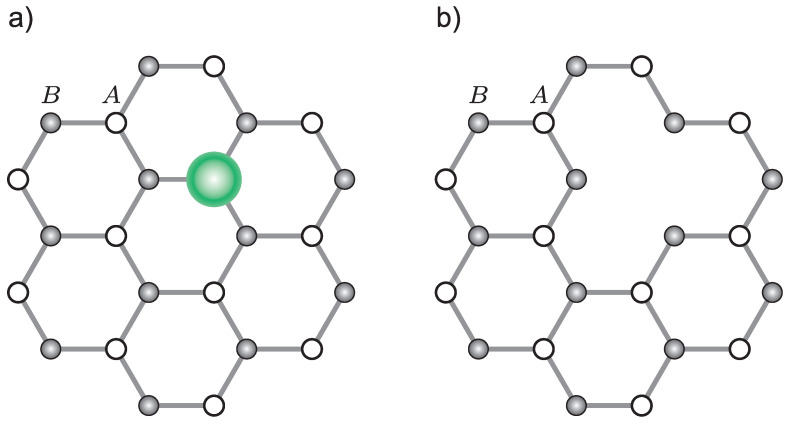
Single-node doping in graphene, (**a**) a new atom instead of a carbon atom, (**b**) a gap in the graphene lattice.

Let us consider the graphene lattice with a single impurity (presented in Figure 2). To find the wave function we solve the Dirac equation for the point *K*

(6) $$\begin{array}{ccc} & & \left({\hat{H}}_{0}\left(r\right)+\hat{V}\left(r\right)\right)\Psi \left(r,\varphi \right)=\varepsilon \;\Psi \left(r,\varphi \right). \end{array}$$

We use the notation proposed by Kane and Mele for the Hamiltonian of non-interacting particles ${\hat{H}}_{0}\left(r\right)$, which has matrix form with complex elements

(7) $$\begin{array}{c} {\hat{H}}_{0}\left(r\right)=-iv\left(\sigma_{x}\tau_{z}\partial_{x}+\sigma_{y}\partial_{y}\right)=-iv\left(\begin{array}{cc} 0 & \frac{\partial }{\partial x}-i\frac{\partial }{\partial y}\\ \frac{\partial }{\partial x}+i\frac{\partial }{\partial y} & 0 \end{array}\right), \end{array}$$

where the double sign in value $\tau_{z}=\pm 1$ corresponds to the states in Dirac points $K(K^{\prime })$, and $\sigma_{\lambda }$ are the Pauli matrices for λ=x,y [20].

The perturbation is described by the matrix $\hat{V}\left(r\right)$, where we include the impurity potential $V_{0}$

(8) $$\begin{array}{c} \hat{V}\left(r\right)=\left(\begin{array}{cc} V_{0}\delta \left(r\right) & 0\\ 0 & 0 \end{array}\right). \end{array}$$

It corresponds to the case of impurity localized at r=0 acting on the states near the Dirac point *K* or $K^{\prime }$.

Most often, the role of dopant is played by a non-carbon atom substituting carbon or by an interstitial atom in the graphene lattice [21]. Any perturbation of the structure lattice can be also considered as a defect responsible for the doping. We assume that impurity induces the same energy levels belonging to different valleys. The distance between the valleys in the inverse space is of the order of $|K-K^{\prime }|∼1/a_{w}$, and the Fourier transform of the matrix Equation (8) V(k) has non-zero elements only at the diagonal. The wave number satisfies inequality $k\ll 1/a_{w}$, and we omit the intervalley transitions because the characteristic range *w* of the potential $V_{0}$, which induces perturbation deployed into the lattice is $w\gg a_{w}$, where $a_{w}∼0.2$ nm. Such a potential does not induce inter-valley scattering. Transitions between valleys are associated with the large momentum which leads to position uncertainty, so we do not consider these transitions. This assumption is justified since the Hamiltonian Equation (3) describes only a small neighbourhood of the Dirac points. Besides, we do not take into account changes in the transition integrals due to the presence or absence of a new atom. This allows to perform calculations for graphene lattice without considering deformations of the graphene structure.

From the Schrödinger equation we obtain the Bessel differential equation [22]

(9) $$\begin{array}{c} \varrho^{2}\frac{d^{2}}{d\varrho^{2}}\psi \left(\varrho \right)+\varrho \frac{d}{d\varrho }\psi \left(\varrho \right)+\left(\tau^{2}-m^{2}\right)\psi \left(\varrho \right)=0, \end{array}$$

where

(10) $$\begin{array}{c} \tau =\frac{\varrho |\varepsilon |}{v}. \end{array}$$

The solutions of this Equation (9) are Bessel functions of first and second kind $J_{\pm \nu }\left(z\right)$, $Y_{\nu }\left(z\right)$, usually called the Weber and Neumann functions, respectively, [22,23]. The functions $J_{\pm \nu }\left(z\right)$ and $Y_{\nu }\left(z\right)$ are regular and holomorphic in the complex plane, and for all values ν are linearly independent. Any linear combination of the Weber and Neumann functions is the solution of Equation (9). By applying for the function χ(ϱ) substitution ν→m, z→τ, we can find function ψ(ϱ) in the form of a linear combination of Bessel functions

(11) $$\begin{array}{c} \psi \left(\varrho \right)=C_{1}J_{m}\left(\tau \right)+C_{2}Y_{m}\left(\tau \right). \end{array}$$

The general solution for the wave function Ψ(ϱ,φ) can be found by considering the form $\psi \left(\varrho ,\varphi \right)∼e^{im\varphi }\psi \left(\varrho \right)$ i $\chi \left(\varrho ,\varphi \right)∼e^{i(m+1)\varphi }\chi \left(\varrho \right)$ and the solution (11). Finally, we obtain the general solution of Equation (9)

(12) $$\begin{array}{c} \Psi_{m}\left(\varrho ,\varphi \right)=\left(\begin{array}{c} e^{im\varphi }\left\{C_{1}J_{m}\left(\frac{\varrho |\varepsilon |}{v}\right)+C_{2}Y_{m}\left(\frac{\varrho |\varepsilon |}{v}\right)\right\}\\ -i\;\mathrm{sgn}\varepsilon \;e^{i(m+1)\varphi }\left\{C_{1}J_{m+1}\left(\frac{\varrho |\varepsilon |}{v}\right)+C_{2}Y_{m+1}\left(\frac{\varrho |\varepsilon |}{v}\right)\right\} \end{array}\right). \end{array}$$

#### Energy of Localized State

Let us consider Equation (6) including the obtained form of wave function (12). We will calculate the energy of state located at impurity at r=0 [24,25]. In the perturbation matrix defined by relation (8) we take into account the potential of defect localized on the sublattice B. It means that we have to solve the system of two equations with the potentials $V_{0}^{A}$, $V_{0}^{B}$ for two corresponding graphene sublattices. The dependence of the resonance energy on the dopant potential is shown in Figure 4. As can be seen its distribution has the character of inverse proportionality. This dependence has a significant influence on the magnetization.

Let us consider the equation

(13) $$\begin{array}{c} \left(-ivs.\left(\sigma_{x}\tau_{z}\partial_{x}+\sigma_{y}\partial_{y}\right)+\hat{V}\left(r\right)-\varepsilon \right)\Psi_{m}\left(\varrho ,\varphi \right)=0, \end{array}$$

where $V\left(r\right)=\mathrm{diag}\{V_{0}^{A}\delta \left(r\right),V_{0}^{B}\delta \left(r\right)\}$. We are looking for solutions that are proportional to the functions $Y_{m}\left(\tau \right)$. We assume that m=0, in addition we put $C_{2}=1$. Using the same assumptions that we made in the case of solution proportional to the function $J_{m}\left(\tau \right)$, we have

(14) $$\begin{array}{c} V_{0}^{A}\;Y_{0}{\left(\frac{\varrho_{0}\left|\varepsilon \right|}{v}\right)}_{\varrho_{0}\to 0}-\int \left(\varepsilon +\mathrm{sgn}\varepsilon \;|\varepsilon |\right)Y_{0}\left(\frac{\varrho_{0}\left|\varepsilon \right|}{v}\right)d^{2}r=0, \end{array}$$

For ε>0 Equation (14) acquires the following form

(15) $$\begin{array}{c} V_{0}^{A}\;Y_{0}{\left(\frac{\varrho_{0}\left|\varepsilon \right|}{v}\right)}_{\varrho_{0}\to 0}-4\pi \varepsilon \int_{0}^{\varrho_{0}}\varrho \;Y_{0}\left(\frac{\varrho_{0}\left|\varepsilon \right|}{v}\right)\;d\varrho =V_{0}^{A}\;\frac{2}{\pi }\ln\left(\frac{\varrho_{0}\left|\varepsilon \right|}{v}\right)+\frac{8v^{2}}{\varepsilon }=0, \end{array}$$

which leads to solution of this equation in the form

(16) $$\begin{array}{c} \varepsilon =\mp \frac{4\pi v^{2}}{V_{0}^{A}\ln\left(\frac{\varrho_{0}\left|\varepsilon \right|}{v}\right)}. \end{array}$$

### 2.2. Magnetization Induced by Impurity

The local magnetization M(r) of graphene can be found by multiplying electron density n(r) by magnetic moment of a single electron $m=g\mu_{B}s$, where g=2.0023 is the Landé factor, and $\mu_{B}=\frac{e\hbar }{2m_{e}}$(e<0) is the Bohr magneton. This leads to expression

(17) $$\begin{array}{c} M\left(r\right)=m\;n\left(r\right)=g\mu_{B}s\;n\left(r\right). \end{array}$$

The spin density can be written as s(r)=sn(r). Spin vector for the single electron s=σ/2 is proportional to the vector composed of Pauli matrices $\sigma =[\sigma_{x},\sigma_{y},\sigma_{z}]$. Here we put ℏ=1. Equivalently, the magnetization M(r) is the magnetic moment of the unit area. The total spin per surface is equal to the integral

(18) $$\begin{array}{c} s_{0}=\int \;d^{2}r\;s\left(r\right)\;. \end{array}$$

The spin density of electron gas can be expressed by the Green’s function of free electrons [26]

(19) $$\begin{array}{c} s\left(r\right)=-i\;\mathrm{Tr}\int_{-\infty }^{\infty }\frac{d\varepsilon }{2\pi }\;\sigma G_{0}\left(\varepsilon ;r,r\right). \end{array}$$

Calculations of the magnetization will be performed in the second order of perturbation. Hamiltonian has the form $\hat{H}={\hat{H}}_{0}+{\hat{H}}_{int}$, where ${\hat{H}}_{0}$ is nonperturbated WDH (3). In addition, the Hamiltonian of the perturbation ${\hat{H}}_{int}$ is connected with the spin of impurity as follows

(20) $$\begin{array}{c} {\hat{H}}_{int}^{m}=g_{c}\mu_{z}\left(r\right)\sigma_{z}, \end{array}$$

where $g_{c}$ is the coupling constant, $\mu_{z}\left(r\right)=g\mu_{B}\;\frac{1}{2}\;{\left|\Psi \left(r\right)\right|}^{2}$ is the magnetic density profile proportional to the probability distribution function of the electron with spin s localized on impurity at the point r=0 in sublattice *A*. The expression (20) describes the interaction of magnetic moment located at impurity with electrons filling the valence band of graphene close to the Fermi energy $E_{F}$ at the Dirac point *K*. When doing so, we assume that the Coulomb interaction is strong enough so that the impurity level is occupied by one electron. In the second order of perturbation we express the full Green’s function

(21) $$\begin{array}{ccc} & & \hat{G}\left(\varepsilon \right)={\left(\varepsilon -\hat{H}\right)}^{-1}, \end{array}$$

by the function ${\hat{G}}_{0}\left(\varepsilon \right)$ of the system of noninteracting particles [26] and the Hamiltonian of interaction ${\hat{H}}_{int}$ [27]. Considering the relations (18) and (19) in the second order of perturbation, we can calculate the total spin projection on the magnetization axis *z*

(22) $$\begin{array}{ccc} s_{z}\left(r\right) & = & -i\;\mathrm{Tr}\;\int_{-\infty }^{\infty }\frac{d\varepsilon }{2\pi }\;\sigma_{z}\;\hat{G}\left(\varepsilon ;r,r\right)\\ & = & -i\;\mathrm{Tr}\;\int d^{2}r^{\prime }\;\int_{-\infty }^{\infty }\frac{d\varepsilon }{2\pi }\;\sigma_{z}\;{\hat{G}}_{0}\left(\varepsilon ;r,r^{\prime }\right){\hat{H}}_{int}{\hat{G}}_{0}\left(\varepsilon ;r^{\prime },r\right). \end{array}$$

To calculate the impurity-induced magnetization $M_{z}\left(r\right)$ we should consider magnetic perturbation ${\hat{H}}_{int}$ at the point $r^{\prime }\neq r$ and single-particle Green’s function of free electrons in graphene. As a result we get

(23) $$\begin{array}{c} M_{z}\left(r\right)=g\mu_{B}\;s_{z}\left(r\right)=-ig_{c}g\mu_{B}\;\mathrm{Tr}\int d^{2}r^{\prime }\;\int_{-\infty }^{\infty }\frac{d\varepsilon }{2\pi }\;\sigma_{z}\;G_{0}\left(\varepsilon ,R\right)\;\mu_{z}\left(r^{\prime }\right)\sigma_{z}\;G_{0}\left(\varepsilon ,-R\right). \end{array}$$

In the above formula, the constant $g_{c}$ is taken positive since the RKKY interaction is ferromagnetic [28,29], and vector $R={r-r}^{\prime }$.

Considering the magnetic profile $\mu_{z}\left(r\right)=\frac{1}{2}g\mu_{B}{\left|\Psi \left(r^{\prime }\right)\right|}^{2}$, which is associated with the localized electron at impurity, we obtain

(24) Mz(r)=gcg2μB216πv∫ζ1(ϱ,ϱ′,φ)d2r′+Im∫ζ2(ϱ,ϱ′,φ)d2r′

where

(25) $$\begin{array}{c} \zeta_{1}\left(\varrho ,\varrho^{\prime },\varphi \right)=\frac{|\Psi \left(\varrho^{\prime }\right){|}^{2}}{2R^{3}}, \end{array}$$

and

(26) $$\begin{array}{c} \zeta_{2}\left(\varrho ,\varrho^{\prime },\varphi \right)=\frac{|\Psi \left(\varrho^{\prime }\right){|}^{2}}{R^{3}}\Lambda \left(r^{\prime },\varphi \right). \end{array}$$

The magnetization depends on the function $\zeta_{1}\left(\varrho ,\varrho^{\prime },\varphi \right)$, decreasing with *R* as ${\left|R\right|}^{-3}$, which can be applied to pure graphene in the ground state, for which the chemical potential and the Fermi energy $\mu =E_{F}=0$, corresponding to the energy at the Dirac point [28]. The effect of free carriers on impurity-induced magnetization is determined by the function $\zeta_{2}\left(\varrho ,\varrho^{\prime },\varphi \right)$.

Let us transform the integral $\zeta_{1}\left(\varrho \right)$ to the form useful for numerical calculations

(27) $$\begin{array}{c} \zeta_{1}\left(\varrho \right)=\int_{0}^{\infty }\frac{\varrho^{\prime }\;{\left|\Psi \left(\varrho^{\prime }\right)\right|}^{2}}{2}d\varrho^{\prime }\;\int_{0}^{2\pi }d\varphi \frac{1}{{\left(\varrho^{2}+{\varrho^{\prime }}^{2}-2\varrho \varrho^{\prime }\cos\;\varphi \right)}^{3/2}}. \end{array}$$

We introduce the function $\vartheta_{1}\left(\varrho ,\varrho^{\prime }\right)=\int_{0}^{2\pi }R^{-3}d\varphi$, which can be expressed by complete elliptic integrals K i E of the first and second kind, respectively, [30],

(28) $$\begin{array}{c} \vartheta_{1}\left(\varrho ,\varrho^{\prime }\right)=-4\frac{d}{da}\frac{2}{\sqrt{a+1}}\;\;K\left(\sqrt{\frac{2}{a+1}}\right)=\frac{4E\left(\sqrt{\frac{2}{a+1}}\right)}{\sqrt{a+1}\left(a-1\right)}, \end{array}$$

where $a=\frac{\varrho^{2}+\varrho^{\prime 2}}{2\varrho \varrho^{\prime }}$. In this way, the solution is obtained in the form

(29) $$\begin{array}{c} \zeta_{1}\left(\varrho \right)=\int_{0}^{\infty }\frac{2\;\varrho^{\prime }{\left|\Psi \left(\varrho^{\prime }\right)\right|}^{2}\;E\left(\frac{2\sqrt{\varrho \varrho^{\prime }}}{\left(\varrho +\varrho^{\prime }\right)}\right)}{\left(\varrho +\varrho^{\prime }\right){\left(\varrho -\varrho^{\prime }\right)}^{2}}d\varrho^{\prime }\;. \end{array}$$

## 3. Results

Graphene without free carriers at T=0 with the Fermi energy $E_{F}=0$ corresponding to the Dirac point, is usually called *intrinsic graphene*. In this case the valence band is fully occupied by electrons and the conduction band is empty [31]. Therefore, in this case we get

(30) $$\begin{array}{ccc} M_{z}\left(\varrho \right) & = & \frac{g_{c}g^{2}\mu_{B}^{2}}{16\pi v}\int_{0}^{\infty }\frac{2\;\varrho^{\prime }\;{\left|\Psi \left(\varrho^{\prime }\right)\right|}^{2}\;E\left(\frac{2\sqrt{\varrho \varrho^{\prime }}}{\varrho +\varrho^{\prime }}\right)}{\left(\varrho +\varrho^{\prime }\right){\left(\varrho -\varrho^{\prime }\right)}^{2}}d\varrho^{\prime }. \end{array}$$

As we see, the magnetization (30) is the function of such parameters as the impurity potential $V_{0}$, the exchange interaction constant $g_{c}$ and the distance ϱ.

**Figure 3:**
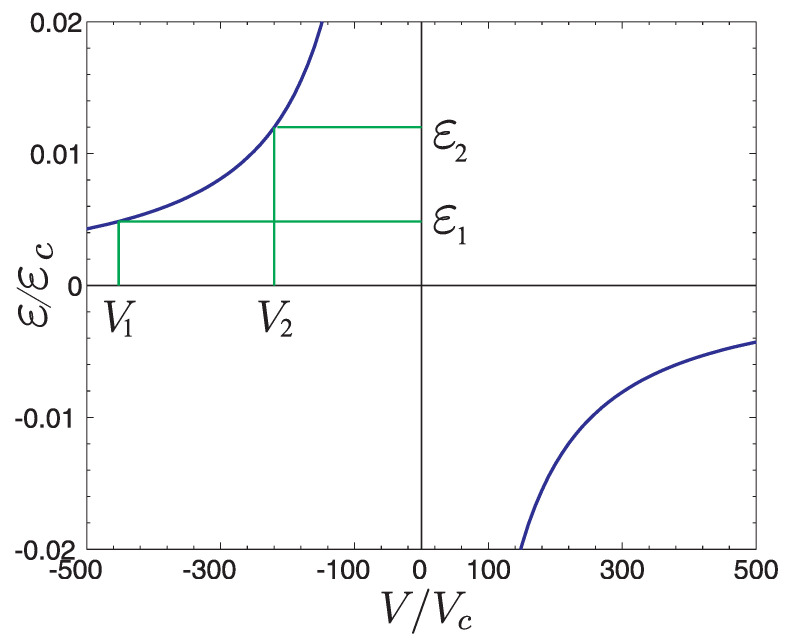
Dependence of resonant energy levels ε of a state localized on a non-magnetic impurity with the potential $V_{0}$ Equation (16), where $\varepsilon_{c}=\hbar^{2}/\left(m_{e}a_{0}^{2}\right)$, $V_{c}=\varepsilon_{c}a_{0}^{2}$, $m_{e}$ is the mass of electron, and $a_{0}$ is the distance between carbon atoms. Localized states without magnetic correlations.

**Figure 4:**
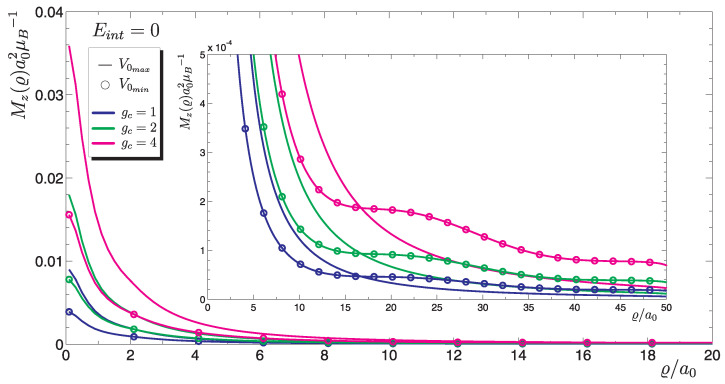
Induced local magnetization $M_{z}\left(\varrho \right)$ as a function of the distance from the impurity center ϱ.

We consider a graphene monolayer sample. At the point $\varrho^{\prime }$ of hexagonal lattice there is a chemical dopant in form of an atom other than C or a vacancy in the structure. The existence of this type of non-magnetic defect with a certain potential $V_{0}$ causes a new level to appear ε (or levels, in case of non-zero spin–orbit interaction) localized (resonance) described by Equation (16) and presented in Figure 3. Numerical solutions shown in Figure 4 contain no contribution from magnetic interaction energy $E_{int}=0$, because the magnetic correlations are not taken into account. If the Coulomb interaction *U* is so large that the doping level can be occupied by only one electron, for which the distribution of magnetic density $\mu_{z}\left(r\right)$ on the plane, is associated with the probability density ${\left|\Psi \left(r\right)\right|}^{2}$, then one can expect the existence of magnetic polarization. Although the impurity is point-like (i.e., located within the sublattice *A*) the electron charge given by ${\left|\Psi \left(r\right)\right|}^{2}$ is spread over a large area $\varrho /a_{0}∼50$ polarizing spins of the valence electrons, for which $E_{F}=\mu =0$.

By analyzing Figure 3 we note that this situation can occur if the Fermi level $E_{F}$ is equal to $E_{F1}$. Then one can expect polarization of spins around the impurity with potential $V_{1}$, corresponding to the energy $\varepsilon_{0}$, and the magnetization distribution is described by expression (30).

As we mentioned, the spin density depends on the distribution function ${\left|\Psi \left(\varrho \right)\right|}^{2}$. In turn, such a state depends on the energy $\varepsilon (V_{0})$. Since the argument of the wave function (12) is the absolute value of the energy of localized state |ε|, the solutions does not depend on the sign of impurity potential. Numerical solutions (Figure 4) contain results for different values of the constant $g_{c}$ (they are represented by the lines distinguished by colors). It is observed that as the electron coupling constant increases, the magnetization of graphene changes. Based on the solutions for $M_{z}\left(\varrho \right)$, it can be assumed that the smallest contribution to the total magnetization $M_{T}$ will give solutions marked with a circle, representing the minimum impurity potential $\pm V_{0_{min}}$. The largest magnetic moment is obtained for potentials $\pm V_{0_{max}}$. We will see this in the next section, where calculations will be presented for the total magnetic moment of an area whose characteristic size is determined by the function ${\left|\Psi \left(\varrho \right)\right|}^{2}$.

As shown by Abrikosov [32] on an example of the InSb structure doped with tellurium, even if the Fermi energy is below the impurity level ε then due to the renormalization of impurity level by local magnetic correlation to the position $\varepsilon_{1}$ the spin polarization can appear. The interaction (correlation) energy will be introduced below, and the mentioned effect will be further described by using the phase diagram as an example.

### Magnetic Correlations—Interaction Energy

In our model we assume that the localized charge on impurity is the reason of spin polarization in the graphene lattice. This polarization is related to the magnetic moment, which is proportional to $\mu_{z}\left(r\right)∼{\left|\Psi \left(\varrho \right)\right|}^{2}$. In this case, the interaction energy of a localized magnetic moment with the polarized band charges should be taken into account. We perform a similar analysis in the case of free charges μ≠0. The magnetic interaction energy in our model can be written in the following form

(31) $$\begin{array}{c} E_{int}=-g_{c}\int d^{2}\;r\;M_{z}{\left(r\right)|\Psi \left(r\right)|}^{2}=-g_{c}2\pi \int_{0}^{\infty }\varrho M_{z}\left(\varrho \right){\left|\Psi \left(\varrho \right)\right|}^{2}\;d\varrho . \end{array}$$

Taking into account the energy $E_{int}$ described by relation (31) leads to renormalization of the impurity level ε. The effect of this interaction on ε illustrates Figure 6.

**Figure 5:**
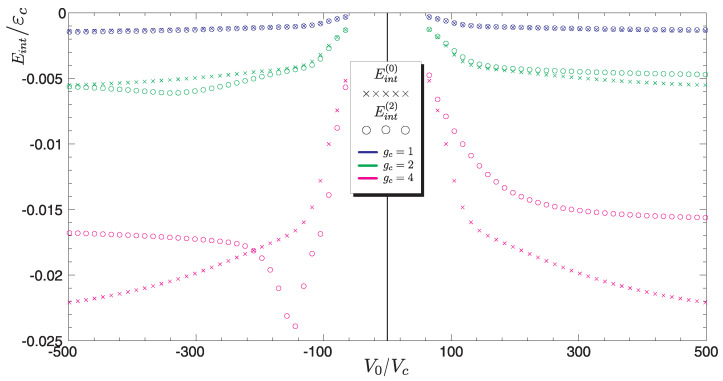
Interaction energy $E_{int}$ as the function of impurity potential $V_{0}$. ×—the results obtained for the interaction energy in the first iteration step (see the blue dashed line (Figure 6)). Solutions marked with a circles correspond to $E_{int}$ in the last iteration step of the self-reconciliation procedure. Colors denote different values of the constant $g_{c}$.

**Figure 6:**
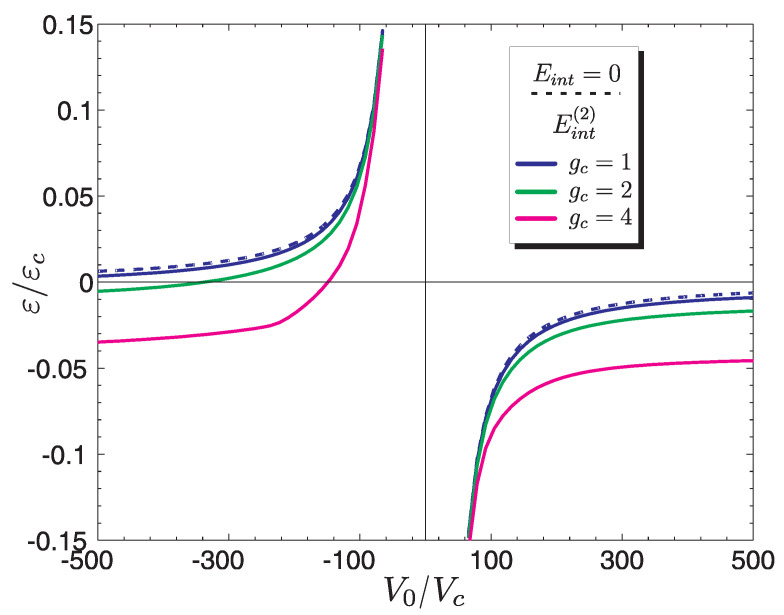
Resonant energy levels ε with consideration of magnetic interaction $E_{int}$ as a function of renormalized impurity potential $V_{0}$ for different values of coupling constant $g_{c}$.

As can be seen in Figure 5 the results marked with (×) represent the interaction energy just after *inclusion* spin polarization for different values of the coupling constant $g_{c}$. In this case, the impurity level in Figure 6 is represented by the dashed blue line. In Figure 6, the effect of magnetic doping interaction is included, compared to Figure 5, in which non-magnetic doping was considered. For these energy levels we obtain the results shown in Figure 4.

The results, which are marked with a circle, correspond to inclusion of the magnetic interaction energy. Let us denote the energy of impurity level after renormalization as $\varepsilon_{\alpha }^{\prime }$. The minus sign in the Formula (31) assumes ferromagnetic interactions between the charges, so we can write

(32) $$\begin{array}{c} \varepsilon_{\alpha +1}^{\prime }=\varepsilon_{\alpha }+E_{int}^{\left(\alpha \right)} \end{array}$$

We should study the convergence of the sequence $\varepsilon_{\alpha }$ to a fixed value, which requires that $\Delta \varepsilon^{\prime }=\varepsilon_{\alpha +1}-\varepsilon_{\alpha }\to 0$. Therefore, this procedure was done for α=0,1,2.

## 4. Summary and Discussion

To date, the magnetic correlations were not properly considered in the doped magnetism of graphene or carbon materials in general. Our results show that they can be of fundamental importance. A separate issue is the effect of RKKY oscillations on the magnetic state. The variation of chemical potential μ results in appearance of free carriers.

Here we discuss the impurity-induced electronic and magnetic properties of graphene, especially the role of impurity in formation of the localized states with resonant energy levels [33,34]. The energy of impurity state depends on impurity potential. It can be found from the Schrödinger equation in the model with Dirac Hamiltonian. We discuss the results using the methods of perturbation theory [35]. As a result we obtain the possibility of impurity-induced magnetization of graphene. We use the model of point perturbation located in one graphene sublattice. The wave function of the state of electron localized on impurity has been also found. It can be presented in form of the Bessel functions.

As a result of calculations we have determined the energy levels of dopant localized states in graphene. We have described the local magnetization induced by the presence of the dopant. We have consider the effect of the potential perturbation induced by the presence of the impurity, and we study the relation between local magnetization and distance from the impurity center. To calculate the results of spin projections on the magnetization axis, we examined the method of Green’s functions. Finally we include the interaction energy of a localized magnetic moments as a function of normalized impurity potential.

The obtained results show a significant and measurable effect of doping on the electron structure and magnetic properties of graphene. These results are in agreement with previous work indicating that doping of graphene can be used to obtain a material with desirable transport properties of both electric charge and spin currents. At the same time, the work done indicates that many properties of graphene need further investigation both on the ground of theory and experiment. Improvement of computational technique using perturbation theory as well as reaching for tools that go beyond perturbation methods seems to be important in further research.
